# Supplementary material for: Mycolactone A vs. B: Multiscale Simulations Reveal the Roles of Localization and Association in Isomer-Specific Toxicity
Source: Toxins (Basel). 2023 Aug 2;15(8):486. doi: 10.3390/toxins15080486 (PMC10467071; doi:10.3390/toxins15080486)
Supplement: Supplementary file 1 [file toxins-15-00486-s001.zip › toxins-2420681-supplementary.pdf]

toxins

MDPI

Supplementary Materials: Mycolactone A vs. B: Multiscale Simulations Reveal the Roles of Localization and Association in Isomer-Specific Toxicity

John D. M. Nguyen, Gabriel C. A. da Hora and Jessica M. J. Swanson

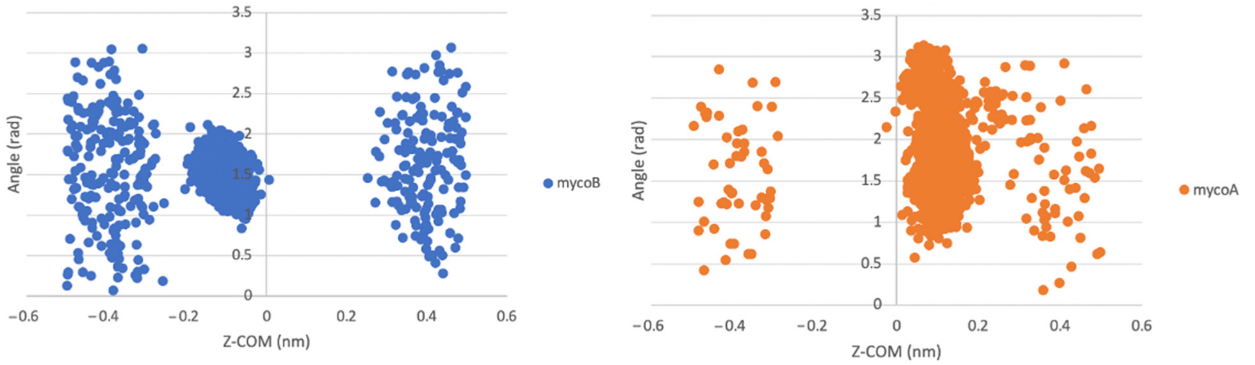

Figure S1. Distribution of the two CVs in unbiased simulations of mycolactone A/B with the ER membrane.

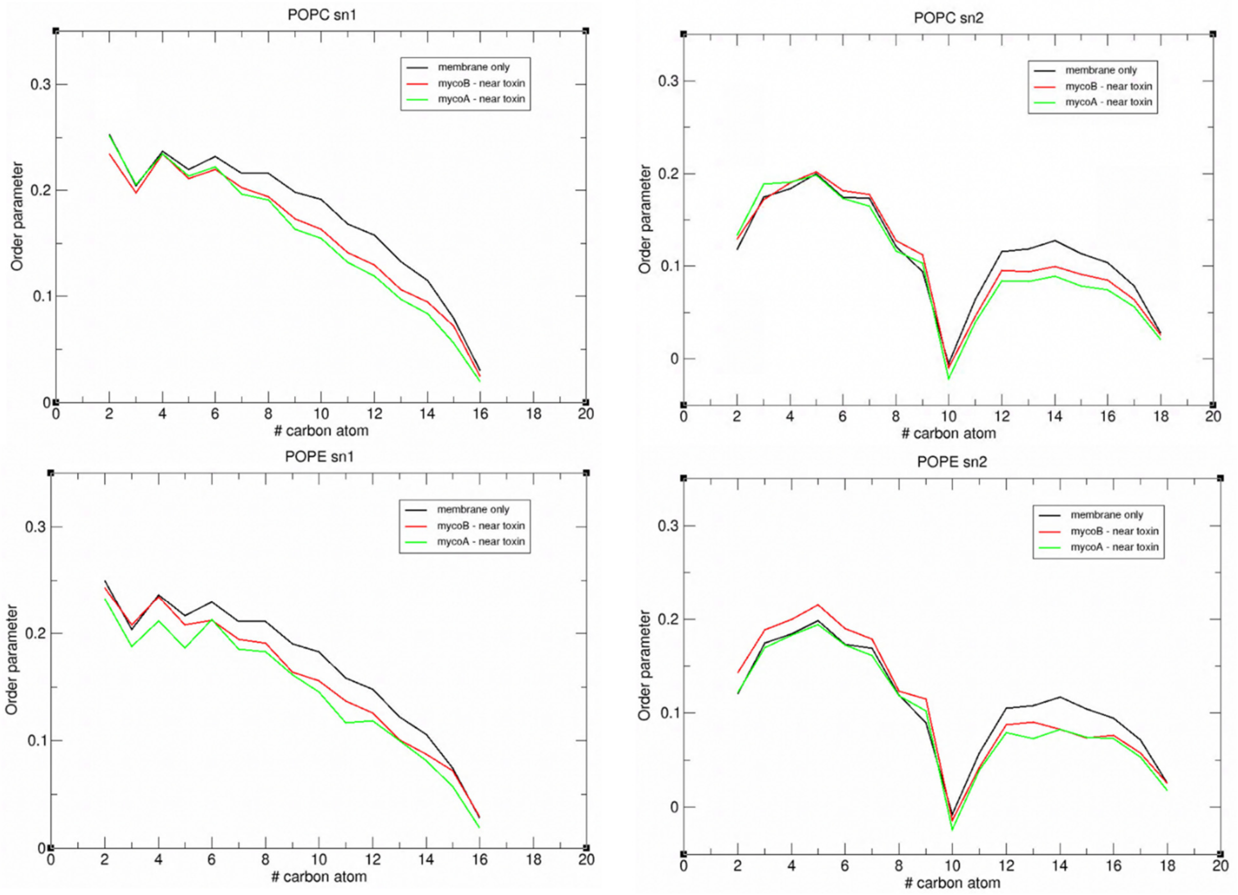

Figure S2. Tail order parameters of lipids near mycolactone and lipids of an ER membrane without the toxin.

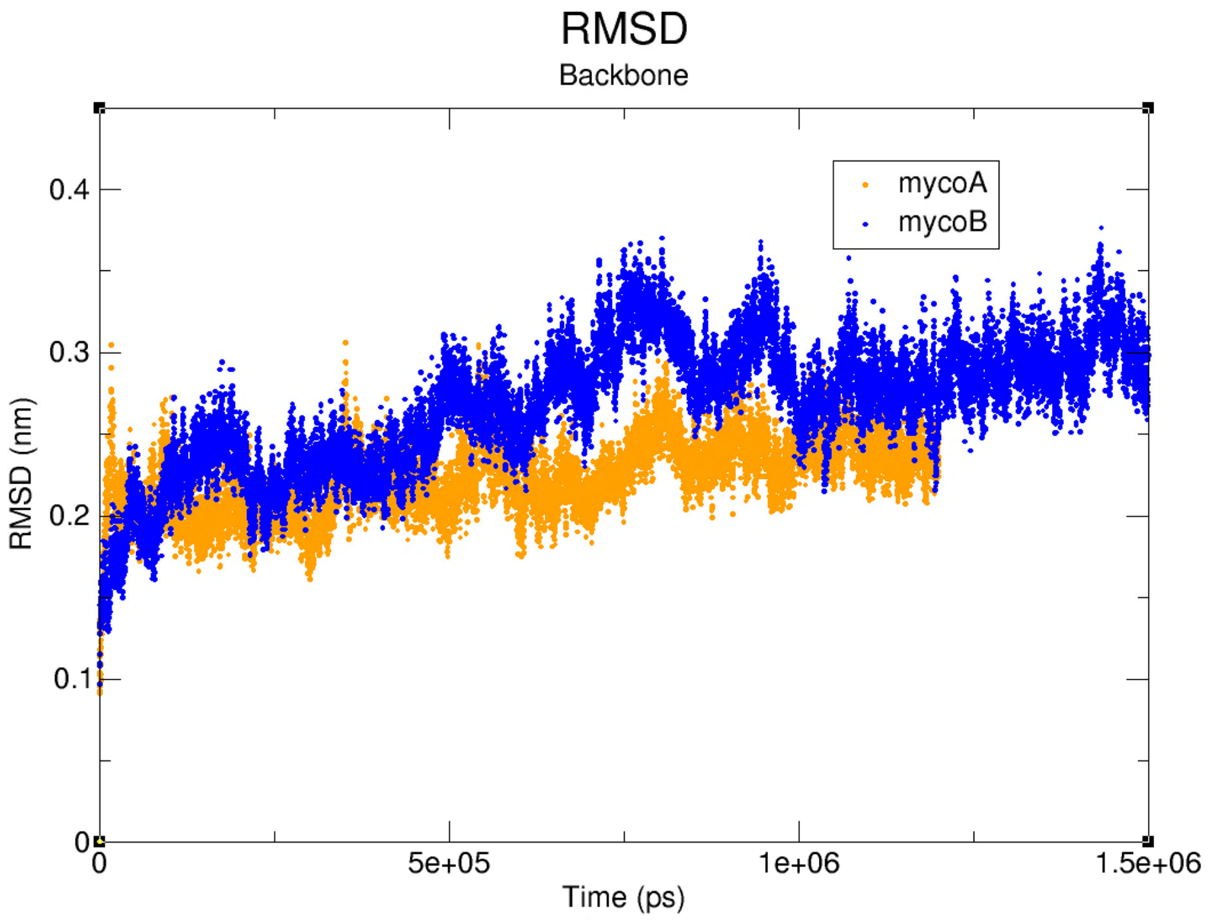

Figure S3. Root mean square deviation of mycolactone-Sec61 complexes.
